# Giant Diaphragmatic Angiosarcoma of Adult: A Case Report and Review of Literature

**DOI:** 10.1155/2012/950856

**Published:** 2012-02-01

**Authors:** Tao Ren, Xue-qian Chen

**Affiliations:** ^1^Department of Oncology, The Affiliated Hospital, North Sichuan Medical College, Nanchong 637000, China; ^2^Key Disciplines of Oncology in Sichuan Province, The Affiliated Hospital, North Sichuan Medical College, Nanchong 637000, China; ^3^Department of Clinical Medicine, North Sichuan Medical College, Nanchong 637000, China

## Abstract

Angiosarcoma is a rare vascular malignant soft tissue tumor, with highly malignant, invasive, and multifocal characteristics of biology, which is prone to local recurrence and distant metastasis, so the prognosis is extremely poor. It rarely involves the diaphragm. We present the case of an adult patient who had a primary giant angiosarcoma of the left-sided diaphragm.

## 1. Introduction

Angiosarcoma is a rare vascular malignant tumor originat-ing from the endothelial cells in small blood vessels, which represents 1-2% of soft tissue tumors [1, 2]. It may interest a variety of organs but usually occurs in the skin, breast, soft tissue, spleen, heart, liver, and skeletal muscles [3]. Clinical report is rare for giant diaphragmatic angiosarcoma of adult truly, so the experiences of its diagnosis and treatment are particularly valuable. We report a case of primary giant angiosarcoma of the left-sided diaphragm and compare our experience with a literature review.

## 2. Case Report

**Figure 1:**
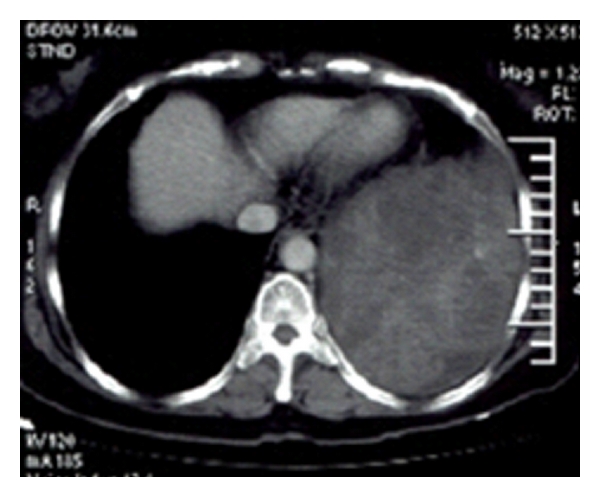
CT findings of angiosarcoma of the diaphragm (tumors were located under the chest, a huge volume, heterogeneous enhancement, No mediastinal lymph nodes).

A 58-year-old woman had a left-sided chest distress and short breath for more than one month duration. No other symptoms such as fever, ague, cough, emptysis, hemoptysis, night sweat, and fatigue were detected. High-density shadow was found in lower left chest after chest film examination, and the further CT revealed a mass opacities with inhomogeneous density and slight enhancement in lower left thoracic cavity, and no mediastinal lymph nodes, as well as a small amount of effusion (see Figure 1). We found a large number of lymphocytes and mesothelial cells by analysing pleural effusion, but no cancer cells or acid-fast bacilli. Meanwhile, CAl25 was significantly increased and reached 452.4 U/mL in tumor marker detection. Comprehensive consideration suggests that the mass opacities in left thoracic cavity may be a tumor. After performing an exploratory thoracotomy under general anaesthesia, the 20 cm × 15 cm × 18 cm lobulated substantial mass, whose basal part was 6 cm × 5 cm and closely linked with segmentum lingulare as well as lower lobe, was confirmed located in left diaphragm. The frozen section of left diaphragm mass was diagnosed as malignant tumor, which is also known as angiosarcoma. Then, tumor was removed entirely.

## 3. Histology and Immunohistology

**Figure 2:**
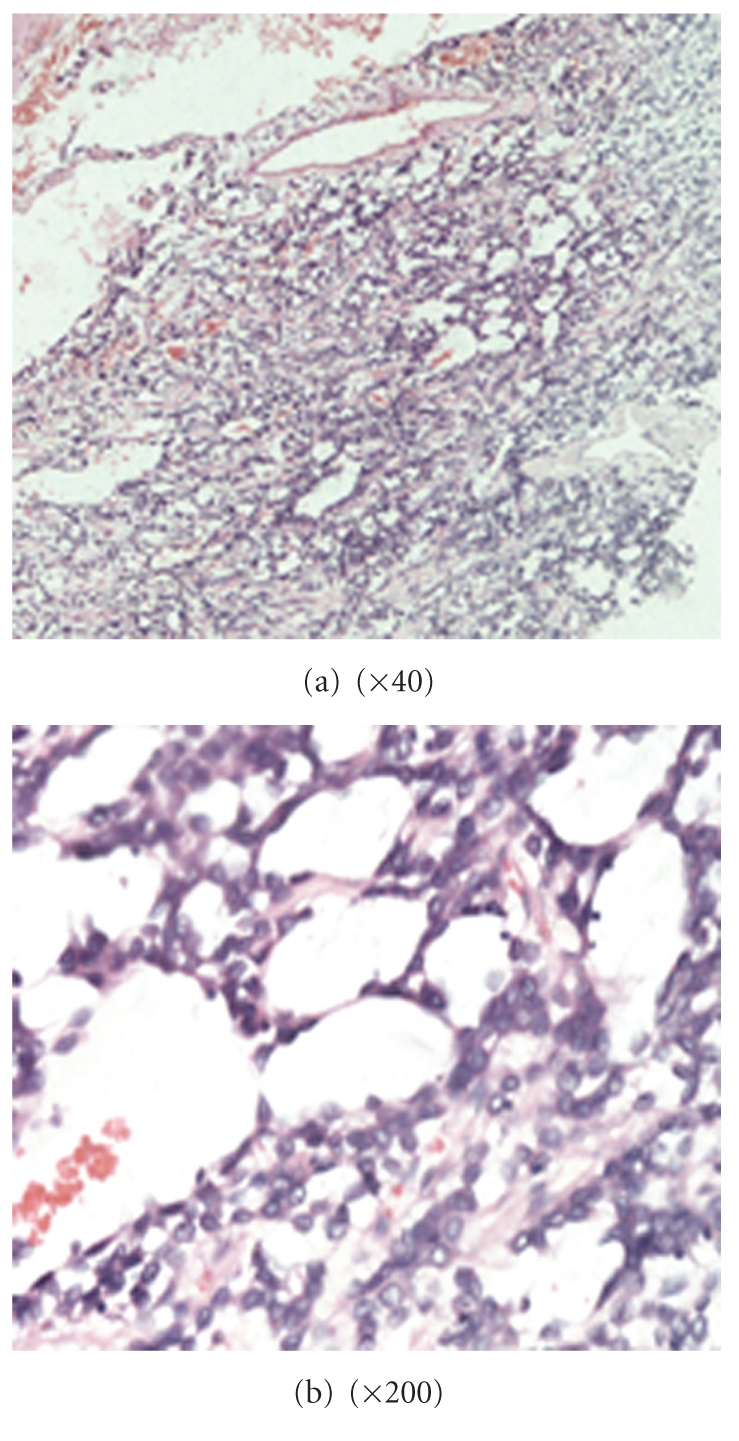
Histopathological microscopic structure of diaphragm angiosarcoma (tumor cell atypia, abnormal vessel-like cavities structure).

**Figure 3:**
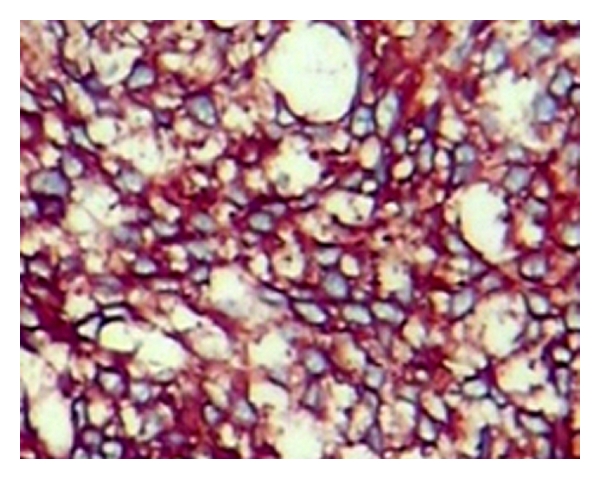
Epithelioid cells strongly positive with CD34 (×200).

Upright fluorescence microscope was used for watch. Biopsy of the lesion showed irregular channels and ecstatic vascular spaces lined by plump hyperchromatic endothelial cells. The histology results were most compatible with moderately differentiated angiosarcoma, as shown in Figure 2, in low magnification (L) and high magnification (R). The diagnosis result was supported by the immunophenotypes CD34 (++) (see Figure 3), CD31 (+), Vimentin (+), epithelial membrane antigen [EMA(−)] phosphoenolpyruvate carboxykinase [PCK(−)], factor VIII related antigen [FVIII (−)], as well.

## 4. Postoperative Radiotherapy and Followup

After surgical treatment, the patient recovery was smoothly. Due to the multifocal and invasive characteristic of angiosarcoma, and combined with limited resection of the left-sided diaphragm, radiotherapy postoperatively 3 weeks later was used. The patient was treated by 3-dimensional conformal radiotherapy (3DCRT), the clinical target including the left side of diaphragm and the part of the lung tissues which adhered to the angiosarcoma. A dose of 60 Gy in 2 Gy fractions was given to 90% isodose volume in 3DCRT. In view of the effect is not clear, postoperative chemotherapy was not used. She was good by following up in outpatient department, and CAl25 was significantly decreased to 63.0 U/mL one month after radiotherapy.

## 5. Discussions

Angiosarcoma with low morbidity and high invasiveness is evolved from endothelial cells or their derivatives of mesenchymal cells. Its incidence may be related to these factors, such as long-term chronic lymphedema, ionizing radiation, chemical exposure, trauma, and chronic infection [4], but the specific mechanic is unclear. Angiosarcoma could occur in any part of the body [3], but the primary angiosarcoma in the diaphragm is rare and there are few clinical reports.

Clinical manifestations of angiosarcoma are diverse, and its clinical features and imaging findings are different from different parts [2, 5]. The patients showed symptoms of chest tightness, shortness of breath for over one month, and CT revealed a huge heterogeneous mass in lower left thoracic cavity. The time of clinical symptoms was not long, but the volume of tumor was huge, and indicated angiosarcoma of diaphragm has the characteristics of occult and fast growing. This may be related to tumor location of the anatomical characteristics, such as the huge volume of the chest, the elastomeric characteristics of lungs and heart; these factors might be conducive to tumor growth and be difficult to be found in the early days.

The diagnosis of angiosarcoma mainly depends on the histomorphological checks, and the immunohistochemistry has great significance for the diagnosis and differential diagnosis of angiosarcoma [5–7]. Vimentin, CD31, CD34, and FVIII factors are the specific makers of tumors from blood vessels. Studies show that when vimentin demonstrates positive, a vascular tumor should be suspected. Further more, when CD31, CD34, and FVIII and other endothelial cells specific makers demonstrate positive, then this supports diagnosis of the angiosarcoma [1, 8, 9]. In this case, the tumor maker of CA125 increased, and, in the course of the operation, it was found that the tumor stemmed from the left diaphragms. In addition to the histological diagnosis and immunohistochemical examinations, we could define the diagnosis of the diaphragmatic angiosarcoma. It is worth noting that the clinical diagnosis of diaphragmatic angiosarcoma requires identification with lung cancer. The author believes that CT features can be used as identification of key points, and angiosarcoma of diaphragm showed the tumor located in the lower left thoracic cavity, rare mediastinal and hilar lymph node metastasis. And lung cancer often has mediastinal or hilar lymph node metastases. Secondly, the clinical manifestations of angiosarcoma of diaphragm are different from lung cancer (such as cough, hemoptysis, expectoration, and chest pain).

There is still no standard therapeutic method of angiosarcoma to follow. Surgical treatment, radiotherapy, chemotherapy, immune therapy, and other comprehensive therapy treatment are often adopted. Surgical excision is the main treatments of angiosarcoma. Radiotherapy or chemotherapy before or after the operation may reduce the local recurrence and metastasis [5, 10, 11]. Owing to the multifocal and invasive characteristic of the angiosarcoma, postoperative adjuvant radiotherapy can help increase the local control rates [5, 12]. In terms of time dose fractionation (TDF), it is suggested that the multifocal and ill-defined angiosarcoma be given the doses of 60*∼*70 Gy in order to achieve a better curative effect [13]. In this case, after the complete resection of the tumors, a 3-dimensional conformal radiotherapy is adopted. A dose of 60 Gy in 2 Gy fractions was given to 90% isodose volume, and the patients react well, with light nausea.

The prognosis of angiosarcoma is bad [2, 5]. The factors which markedly effect its prognosis include (1) the initial condition of the patients, namely, that the curative effect of primary cases is significantly better than that of the recurrent or metastatic cases [14]; (2) operation method and operation quality [14, 15], the curative effect of the patients who have the radical operation is better; (3) the tumor size may also influence the prognosis. Studies show that when the tumor diameter is larger than 10 cm, the prognosis is poorer than that when less than 10 cm [16]. The tumor of this case is huge, larger than 10 cm. However, because of its short follow-up time, its survival time and quality of life are still under the follow-up observation.
